# Association of Empirical Dietary Inflammatory Potential with Mortality: Results from the Third National Nutrition Examination Survey

**DOI:** 10.34172/jrhs.2023.113

**Published:** 2023-06-29

**Authors:** Mohamed A. Mostafa, Travis Skipina, Muhammad Ali Anees, Elsayed Z. Soliman, Muhammad Imtiaz Ahmad

**Affiliations:** ^1^Epidemiological Cardiology Research Center, Department of Epidemiology and Prevention, Wake Forest School of Medicine, Winston-Salem, North Carolina, United States; ^2^Department of Internal Medicine, Wake Forest School of Medicine, Winston-Salem, North Carolina, United States; ^3^Department of Internal Medicine, Texas Tech University, Amarillo, Texas, United States; ^4^Department of Internal Medicine, Section on Hospital Medicine, Medical College of Wisconsin, Wauwatosa, Wisconsin, United States

**Keywords:** Inflammation, Diet, Mortality, NHANES

## Abstract

**Background:** The empirical dietary inflammatory potential (EDIP) score is designed to assess the inflammatory potential of a diet based on the pro- and anti-inflammatory properties of its various components. This study examined the association of EDIP with all-cause mortality in a large, community-based, multiracial sample of the United States population.

**Study Design:** A prospective cohort study.

**Methods:** This analysis included 13155 participants (44.6±18.4 years, 54.21% women, and 40.33% White) without prior history of cardiovascular disease (CVD) from the Third National Health and Nutrition Examination (NHANES III) Survey. A 24-hour dietary recall information was used to calculate EDIP. The National Death Index was employed to identify the date and cause of death. Cox proportional hazard analysis was utilized to evaluate the association between the tertiles of EDIP and all-cause mortality over a median follow-up of 26.6 years.

**Results:** In a model adjusted for demographics and CVD risk factors, a higher EDIP tertile, compared with the lowest tertile, was associated with an increased risk of all-cause mortality (hazard ratio [HR]=1.10; 95% CI: 1.02, 1.19). A standard-deviation increase in EDIP (0.27 units) was related to a 4% increased risk of mortality (HR=1.04; 95% CI: 1.01, 1.08). This association was stronger in older participants compared to younger ones (HR=1.09; 95% CI: 0.98, 1.21 vs. HR=0.89; 95% CI: 0.80, 1.01), respectively, interaction *P*=0.030).

**Conclusion:** Pro-inflammatory diet is associated with an increased risk of mortality, especially in the older population. Dietary changes that reduce inflammation may have the potential to reduce the risk of poor outcomes.

## Background

 Inflammation is a common contributor to many metabolic diseases and chronic health conditions associated with disability and mortality.^1,2^ The association of systematic inflammatory biomarkers such as interleukin (IL)-6, C-reactive protein (CRP), and tumor necrosis factor-α (TNF-α) receptor 2 (TNFα-R2) with mortality has been reported in previous studies.^3,4^ Diet is a modifiable risk factor for many health conditions and is an established factor in modulating the levels of these inflammatory mediators.^5,6^ The empirical dietary inflammatory potential (EDIP) is a composite score intended to measure the inflammatory potentials of the whole diet based on the circulating levels of IL-6, CRP, and TNFα-R2^7^. The EDIP score has been used to examine the inflammatory effects of diet on many health conditions such as cancer,^8,9^ autoimmune diseases, and cardiovascular disease (CVD).^10,11^ The association of the inflammatory effects of diet with mortality using the EDIP score and other similar scores such as the dietary inflammatory index (DII) has been reported previously.^12-15^ Nevertheless, two of the cornerstones of scientific advancement are rigor in designing scientific research and the ability to reproduce research findings. The application of rigor and reproducibility ensures the robust and unbiased interpretation of results. In this study, we highlight the significance of identifying dietary factors that can regulate inflammation, as it can have vital implications in reducing the burden of chronic diseases and promoting overall population health. It reinforces the fact that diet is a modifiable risk factor, and adopting healthy eating patterns is crucial for preventing and managing chronic diseases. Additionally, the focus of the study on mortality as an outcome measure further emphasizes the importance of these findings, as all-cause mortality is a meaningful endpoint for assessing the impact of dietary factors on health outcomes. Therefore, it was aimed to examine the association of the EDIP score with overall mortality using the extended duration of follow-ups among participants enrolled in the Third National Health and Nutrition Examination Survey (NHANES-III) from 1988 to 1994 and followed up to December 2019.

## Methods

###  Study population

 The NHANES-III is one of a series of health surveys designed to assess the health and nutritional status of the non-institutionalized United States (US) population using both subjective and objective data through interviews and subsequent appointments at a mobile examination center (MEC). Details of the survey design have been published previously.^16^ Ethical approval was obtained from the National Center for Health Statistics (NCHS) Research Ethics Review Board, and written consent was obtained from all participants.

 Participants aged 18 years or older with available 24-hour dietary recall information who were free from CVD with available mortality outcomes, diet, and relevant covariates were included in this study. However, individuals with extreme energy intake data, missing mortality data or missing dietary, or other relevant covariate data were excluded from the investigation. After all exclusions, 13 155 participants were included in the final analysis.

###  Empirical dietary inflammatory potential score and dietary assessment

 Dietary information was obtained from in-person 24-hour dietary recall data with the use of a personal computer-based, automated, interactive data collection and coding system conducted during in-home interviews and MEC visits during the survey. Each food item was multiplied by its corresponding coefficient to create an EDIP score for each participant. The mentioned score ranges from -5.98 (a maximum anti-inflammatory diet pattern) to + 8.14 (a maximum pro-inflammatory diet pattern), with higher (more positive) scores indicating more pro-inflammatory diets while lower (more negative) scores representing anti-inflammatory diets.

 Details of the calculation of the EDIP are described in the following sections.^7^ Briefly, stepwise linear regressions were used to identify 18 food groups, including fish, tomatoes, processed meats, high-energy beverages, other vegetables, red meats, low-energy beverages, refined grains, organ meats, pizza, wine, leafy green vegetables, dark yellow vegetables, beer, coffee, fruit juice, snacks, and tea. They were most predictive of plasma inflammatory biomarkers such as IL-6, CRP, and TNFαR2. The weighted sum of each of these food groups was employed to create the EDIP score. For the current analysis, the tertiles of EDIP were created to examine its association with the outcome.

###  Assessment of covariates

 Age (in years), gender (men and women), race/ethnicity (White, Black, Mexican American, and others), and smoking status (never, current, and former) were self-reported by standardized questionnaires via home interviews and during the participant’s visit to MEC. Body mass index (BMI) was calculated from height and weight measurements. Diabetes was defined as self-report diabetes or hemoglobin A1c values ≥ 6.5%, or use of diabetes-related medications. The blood samples were collected via venipuncture by a phlebotomist. The samples were analyzed for total cholesterol, triglycerides, glucose, and the like using laboratory procedures as reported by NCHS.^16^

###  Mortality ascertainment

 The endpoint for this study was all-cause mortality ascertained by NCHS using death certificates. The de-identified and anonymized data of the NHANES III participants were linked to NDI Mortality Files with a probabilistic matching algorithm to determine mortality status using the NHANES III sequence number. The NCHS public-use linked mortality file provides mortality follow-up data from the date of NHANES III survey participation up until December 31, 2019 (1988–2019).^17^ Participants with no matched death record were considered to be alive during the entire follow-up period.

 All cause-mortality in the current analysis included all the specified causes of death recorded in the public-use linked mortality files. Cause-of-mortality coding for all US mortality occurring prior to 1999 was determined using the Ninth Revision of the International Classification of Diseases (ICD-9), while all mortality after 1998 follows the Tenth Revision of the International Classification of Diseases, (ICD-10) for mortality occurring in or after 1999. To facilitate and assist researchers with analyses, the NCHS recorded all mortality occurring prior to 1999 coded under ICD-9 guidelines into comparable ICD-10 according to the underlying cause of mortality groups.^17^

###  Statistical analysis

 The characteristics of the participants were stratified by EDIP score tertiles. Univariate analyses were conducted to assess the statistical significance of differences in percentages and means for categorical and continuous variables using the chi-square test and the analysis of variance, respectively.

 Multivariable Cox proportional hazard analysis was used to estimate the hazard ratio (HR) and 95% confidence intervals (CIs) for all-cause mortality for mortality across EDIP tertiles (reference group; First tertile as the most anti-inflammatory/least pro-inflammatory diet) and each increase in one standard deviation in EDIP. Models were adjusted as follows:

 Model 1 was adjusted for age, gender, and race. In addition, Model 2 was adjusted for variables in Model 1 plus diabetes, smoking status, total cholesterol, BMI, systolic blood pressure, antihypertensive agents, and lipid-lowering agents.

 The potential effect modification of the association between the EDIP score and mortality was examined in subgroup analysis stratified by gender (men and women), race (white and non-white), and age groups ( < 65 years and ≥ 65 years) with the calculation of interaction *P* value.

 Additionally, a restricted cubic spline model was employed to examine the graphical relation between the EDIP score and the mortality to assess the potential for a nonlinear association and incorporated knots at the fifth, 50th, and 95th percentiles. A likelihood ratio test was computed to test for linearity in the relation between the EDIP score and mortality.

 All data analyses were performed by SAS (Version 9.4, SAS Institute Inc, Cary, NC), and two-sided *P* < 0.05 indicated statistical significance.

## Results

 A total of 13,155 participants (44.6 ± 18.4 years, 54.21% women, and 40.33% White) were included in this analysis. EDIP scores ranged from -0.14 (most anti-inflammatory) to + 0.46 (most proinflammatory) with a mean of 0.15 EDIP score units. Participants in the 3^rd^ tertile of EDIP were more likely to be young, men, and non-White (Table 1).

**Table 1:** Population chrachterstics of the EDIP

**Characteristics**	**EDIP score tertiles**						***P *** **value**
	**1st Tertile, n=4359**		**2nd Tertile, n=4519**		**3rd Tertile, n=4277**		
	**Number**	**Precentage**	**Number**	**Precentage**	**Number**	**Precentage**	
Men	1872	42.9	2000	44.2	2151	50.2	0.001
Whites	2433	55.8	1729	28.2	1143	26.7	0.001
Hypertension	510	11.7	491	10.8	324	7.5	0.001
Diabetes	283	6.4	386	8.5	303	7.0	0.001
Current smoker	1002	22.9	1112	24.6	1211	28.3	0.001
Obesity	915	20.9	1193	26.4	1122	26.2	0.001
**Characteristics**	**Mean**	**SD**	**Mean**	**SD**	**Mean**	**SD**	***P *** **value**
Total cholesterol	206	44	204	45	119	44	0.001
EDIP score	-0.14	0.14	0.14	0.06	0.46	0.15	0.001
Age (y)	48.9	18.80	45.70	19.20	40.70	17.90	0.001

*Note*. EDIP: Empirical dietary inflammatory potential score; SD: Standard deviation; Obesity: Body mass index ≥ 30 kg/m^2^; Hypertension: Systolic blood pressure (BP) ≥ 130 mm Hg or diastolic BP ≥ 85 mm Hg or the use of antihypertensive medications.Diabetes: Fasting plasma glucose ≥ 126 mg/dL, hemoglobin A1c values ≥ 6.5%, or previous use of diabetes-related medications.

**Figure 1:**
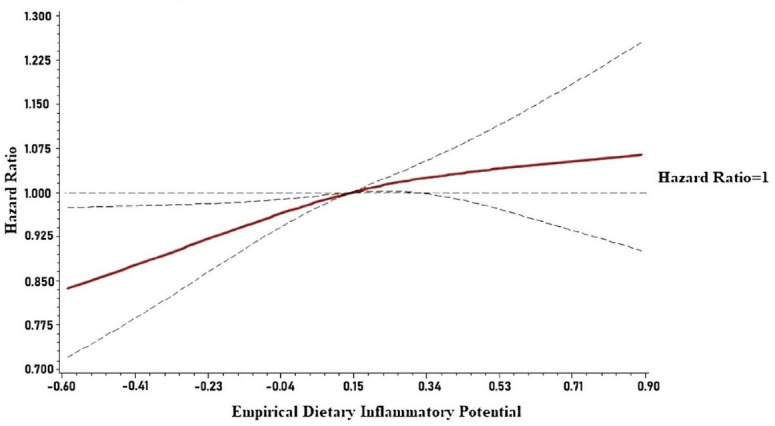
Risk of all-cause mortality by empirical dietary inflammatory potential.
*Note.* EDIP: Empirical dietary inflammatory potential. Each hazard ratio was generated with a median value of the EDIP of 0.14 as a reference and was adjusted for age, gender, race, diabetes, current smoker, total cholesterol, body mass index, systolic blood pressure, antihypertensive medications, and lipid-lowering agents

 Overall, 4840 all-cause deaths occurred over a median follow-up of 26.6 years (IQR: 19.4, 28.6). In models adjusted for demographics and other risk factors, including diabetes, smoking status, total cholesterol, obesity, hypertension, and lipid-lowering agents, higher levels of EDIP were associated with an increased risk of all-cause mortality. Participants with EDIP in the 3^rd^ tertile had a 10% increased risk of mortality (HR = 1.10; 95% CI: 1.02, 1.19) compared to those in the 1^st^ tertile. In a similar model, we investigated the association between each one-unit standard-deviation increase in EDIP and all-cause mortality in a continuous fashion. Each standard-deviation increase in EDIP (0.27 units) was associated with a 4% increased risk of mortality (HR = 1.04; 95% CI: 1.01, 1.08), suggesting a linear association between the two (Table 2). The graphical representation of the risk of mortality across each EDIP value is depicted in Figure 1. As shown, there is a linear trend between increasing the EDIP score and all-cause mortality (*P* value for non-linearity = 0.38).

**Table 2:** Association of Empirical Dietary Inflammatory Potential Score With All-cause Mortality

**EDIP score tertiles**	**Event rate per 1000 person-years**	**Model 1**		**Model 2**	
		**HR (95% CI)**	***P *** **value**	**HR (95% CI)**	***P *** **value**
1^st^ tertile	17.0	Ref.		Ref.	
2^nd^ tertile	15.7	1.09 (1.01, 1.18)	0.002	1.04 (0.98, 1.12)	0.172
3^rd^ tertile	12.3	1.17 (1.07, 1.27)	0.001	1.10 (1.02, 1.19)	0.006
Per 1 standard deviation	14.9	1.06 (1.03, 1.10)	0.003	1.04 (1.01, 1.08)	0.001

*Note*. EDIP: Empirical dietary inflammatory potential score; HR: Hazard ratio; CI: Confidence interval. Model 1 was adjusted for age, gender, and race. Model 2 was adjusted for model 1 plus diabetes, current smoker, total cholesterol, obesity, hypertension, and lipid-lowering agents.

 In subgroup analysis, significant heterogeneity was observed with the strong association of EDIP tertiles with mortality among older participants compared to younger participants (interaction *P* = 0.030). No effect modifications were found by gender (men and women) or race (white and non-white) (Table 3).

**Table 3:** Subgroup analysis of the association between EDIP and all-cause mortality

**EDIP tertiles**	**HR (95% CI)**	***P *** **value**	**Interaction ** ***P *** **value**
Gender			0.181
Men			
2^nd^ Tertile	1.09 (0.99, 1.20)	0.061	
3^rd^ tertile	1.06 (0.96, 1.17)	0.246	
Women			
2^nd^ tertile	1.00 (0.91, 1.09)	0.981	
3^rd^ tertile	1.18 (1.06, 1.31)	0.002	
Race			0.213
Whites			
2^nd^ tertile	1.07 (0.97, 1.17)	0.134	
3^rd^ tertile	1.21 (1.09, 1.34)	0.002	
Non-Whites			
2^nd^ tertile	1.00 (0.91, 1.11)	0.887	
3^rd^ tertile	0.96 (0.86, 1.07)	0.478	
Age group			0.003
< 65 years			
2^nd^ tertile	0.92 (0.84, 1.02)	0.147	
3^rd^ tertile	0.89 (0.80, 1.01)	0.061	
≥ 65 years			
2^nd^ tertile	1.10 (1.00, 1.25)	0.037	
3^rd^ tertile	1.09 (0.98, 1.21)	0.107	

*Note.* Empirical dietary inflammatory potential; HR: Hazard ratio; CI: Confidence interval. Model adjusted for age, gender, race, hypertension, diabetes, current smoker, obesity, total cholesterol, and lipid-lowering agents.

## Discussion

 In this analysis from the US population, it was found that EDIP, an empirically developed tool to assess the inflammatory potential of diet, is significantly associated with the increased risk of all-cause mortality. This association was stronger among older participants compared with younger participants. These findings further validate prior studies that a pro-inflammatory diet is associated with poor outcomes, including mortality.^15,18^

 Previous studies have reported a positive association between high pro-inflammatory diets and mortality.^12-15,18-20^ These studies investigated the relationship between the inflammatory potential of the diet and the risk of mortality in various populations, and the results are consistent with our findings. For instance, Li et al^12^ reported a 26% increased risk of all-cause mortality in a Chinese population, with a stronger association observed among individuals with lower BMI levels. Additionally, Park et al^13^ and Garcia-Arellano et al^14^ found a similar trend between dietary inflammatory potential and all-cause mortality, with a 26% and 22% increased risk, respectively, among Korean and Spanish populations. However, there have been conflicting results regarding the effect modification of this association based on smoking status, BMI levels, and age groups. Therefore, further research is required to examine the observed findings in different populations.

 These studies used DII which involved a retrospective review of the scientific literature to link 45 different food parameters with their inflammatory potential through six inflammatory markers (CRP, IL-1β, IL-4, IL-6, IL-10, and TNF-α).^21^ However, the dietary intake used to calculate DII mainly focused on nutrients rather than whole food items; therefore, the DII can potentially be influenced by nutritional supplements that are not taken into consideration. Hence, its association with mortality may not be completely driven by diet. On the other hand, the EDIP score is an empirically derived metric score with an established association with the serum levels of inflammatory markers. Both EDIP and DII scores have been validated to significantly predict inflammation markers in several populations, with EDIP showing a greater ability to predict the concentrations of plasma inflammatory markers.^22^

 Various healthy dietary patterns such as the healthy eating index, which is derived from adherence to US Dietary guidelines,^23^ and the cultural patterns of diet intake such as the Mediterranean diet mainly consisting of anti-inflammatory diets (i.e., whole grains, green leafy vegetables, fruits, and fish) have been linked to a reduction in the risk of mortality by decreasing the risk and progression of different metabolic diseases due to the overall anti-inflammatory effect of these diets.^15,24,25^ In addition, multiple prior studies have examined the associations of individual dietary items such as red meat or specific nutrient intake such as magnesium or vitamin E with mortality.^18,26,27^ However, one of the limitations of studies focusing on individual foods or nutrients is the consumption of other food items or nutrients which can potentially lead to dietary intercorrelations and may attenuate or accentuate the actual effects of the studied individual food or nutrients.^18^ Therefore, dietary scores such as DII or EDIP may have an advantage as they take into account the diet as a whole incorporating the pro-inflammatory and anti-inflammatory potential of multiple food items taken by an individual.

 In this study, a significant effect modification by age was observed with a stronger association between EDIP and mortality in older participants compared to younger participants. These findings contrast with a previous report in which the association between EDIP and mortality was stronger among those younger than 65 years in comparison to older participants.^12,13,15^ This could be explained by the cumulative effects of pro-inflammatory diets and other factors that induce inflammation as they lived longer. Furthermore, the higher prevalence and duration of chronic diseases in this age group add to the overall inflammatory burden. One possible explanation is the concept of immunaging, which refers to chronic low-grade inflammation that increases with age, and immunosenescence, which is an age-related decline in immune function.^28^ Future studies should explore the potential interplay between participants’ age and dietary inflammatory properties for risk stratification.

 The results of our study should be considered in the context of certain limitations. First, the dietary data were assessed using single 24-hour recall data which may not be a true reflection of participants’ dietary habits over a long period of time; although some studies have shown that dietary patterns are modestly stable over time.^29,30^ Second, although we have adjusted for potential confounders, residual confounding remains a possibility. Finally, dietary recall data, as well as smoking and prior history of CVD, are self-reported and thus subject to recall bias. On the other hand, the strengths of this study include a large, racially diverse, and representative sample of US adults and a long mortality follow-up.

 Based on the association between the inflammatory potential of a diet and overall mortality found in our study, adopting an anti-inflammatory dietary pattern may be beneficial in reducing the risk of mortality. This has significant implications for healthcare providers, as promoting an anti-inflammatory diet may be an effective approach for improving overall health outcomes while reducing the burden of chronic diseases. Similarly, community and public health efforts can focus on promoting an anti-inflammatory dietary pattern to improve health outcomes. Eventually, emphasizing the importance of a plant-based diet and reducing the intake of processed and animal-based foods may be useful in promoting an anti-inflammatory diet.

**Highlights**

- DIP is a validated score that assesses the diet’s inflammatory potential.
- A pro-inflammatory diet increases mortality risk.
- A linear trend exists between EDIP score and all-cause mortality.
- Stronger association exists among older participants.
- Promoting an anti-inflammatory diet improves health and reduces mortality risk.

## Conclusion

 The consumption of a diet with pro-inflammatory properties, estimated by EDIP, is associated with an increased risk of mortality. Advocating for dietary patterns with anti-inflammatory properties may improve overall health, enhance longevity, and reduce the risk of mortality.

## Acknowledgements

 There were no further contributions to this project beyond those of the listed authors.

## Authors’ Contribution

**Conceptualization: **Elsayed Soliman, Muhammad Imtiaz Ahmad.

**Data curation: **Muhammad Imtiaz Ahmad.

**Formal analysis: **Travis Skipina, Muhammad Imtiaz Ahmad.

**Investigation:** Mohamed A. Mostafa.

**Methodology: **Elsayed Z. Soliman.

**Resources:** Muhammad Ali Anees, Mohamed A. Mostafa.

**Validation: **Muhammed Ali Anees.

**Visualization:** Muhammad Imtiaz Ahmad.

**Writing–original draft: **Mohamed A. Mostafa

**Writing–review & editing: **Elsayed Z. Soliman, Muhammad Imtiaz Ahmad.

## Competing Interests

 The authors have no relevant financial or non-financial interests to disclose.

## Funding

 This study received no funds, grants, or other support.
